# Transport spectroscopy of coupled donors in silicon nano-transistors

**DOI:** 10.1038/srep06219

**Published:** 2014-08-28

**Authors:** Daniel Moraru, Arup Samanta, Le The Anh, Takeshi Mizuno, Hiroshi Mizuta, Michiharu Tabe

**Affiliations:** 1 Research Institute of Electronics, Shizuoka University, 3-5-1 Johoku, Naka-ku, Hamamatsu 432-8011, Japan; 2 School of Materials Science, Japan Advanced Institute of Science and Technology, 1-1 Asahidai, Nomi 923-1292, Japan; 3 Nano Research Group, ECS, Faculty of Physical Sciences and Engineening, University of Southampton, Highfield, Southampton SO17 1BJ, United Kingdom

## Abstract

The impact of dopant atoms in transistor functionality has significantly changed over the past few decades. In downscaled transistors, discrete dopants with uncontrolled positions and number induce fluctuations in device operation. On the other hand, by gaining access to tunneling through individual dopants, a new type of devices is developed: dopant-atom-based transistors. So far, most studies report transport through dopants randomly located in the channel. However, for practical applications, it is critical to control the location of the donors with simple techniques. Here, we fabricate silicon transistors with selectively nanoscale-doped channels using nano-lithography and thermal-diffusion doping processes. Coupled phosphorus donors form a quantum dot with the ground state split into a number of levels practically equal to the number of coupled donors, when the number of donors is small. Tunneling-transport spectroscopy reveals fine features which can be correlated with the different numbers of donors inside the quantum dot, as also suggested by first-principles simulation results.

Individual dopant atoms start playing a detrimental role in the operation of downscaled transistors because, since their position and number cannot be precisely controlled, the transistor characteristics will significantly vary from device to device^1^,^2^,^3^,^4^. However, from another approach, it is now possible to address individual dopant atoms in nano-channels by tunneling transport. This ability opened a new field of research, labeled as single-dopant-atom devices, in which one dopant atom (or only a few dopant atoms) plays the role of a quantum dot (QD) in transport. Tunneling transport via individual dopant atoms in silicon transistors has originally been reported in devices with dopants accidentally diffused into the channel^5^,^6^,^7^. Later, in other studies, transport through dopants purposely introduced in the channel was also reported^8^,^9^,^10^,^11^. However, in all these studies, position and number of dopants in the channel remained largely uncontrolled. Only recently, state-of-the-art techniques (scanning tunneling microscope (STM) atom manipulation^12^ and single-ion-implantation^13^) have been used to demonstrate proof-of-concept devices with precisely position-controlled dopants. In these studies, however, dopant position and number in the final device structure are not fully confirmed. Furthermore, these techniques have several restrictions, such as special surface treatment for STM, and they are generally not applicable to practical devices. For practical applications, simpler fabrication techniques must be developed.

In this paper, we report a selective doping technique that allows the formation of QDs containing a few strongly-coupled dopants. This technique uses standard processes, such as electron-beam (EB) lithography patterning combined with thermal-diffusion doping. We also present drastic changes of *I*-*V* characteristics for doped-channel devices with different doping concentrations, i.e., different inter-donor coupling regimes. Low-temperature *I*-*V* characteristics exhibit signatures of electron tunneling transport via a discrete energy spectrum, with the number of resolved levels correlated with the number of coupled donors in a range of a small number of donor-atoms.

## Results

### Nano-channel transistors with isolated donors and with coupled donors

**Figure 1:**
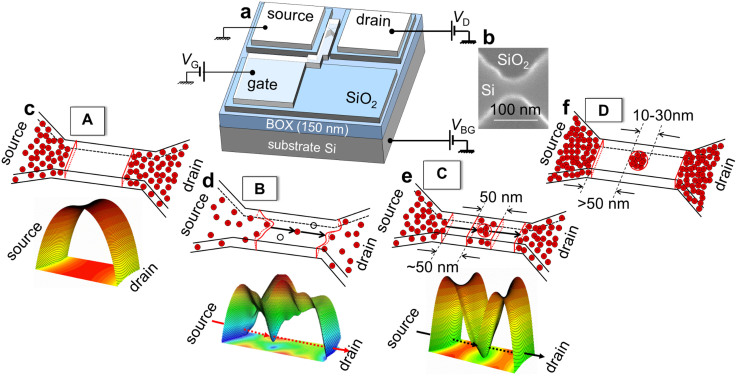
Nanoscale silicon-on-insulator field-effect transistors with different channel-doping profiles. (a), SOI-FET device structure and measurement setup. (b), An SEM image of a typical sub-100-nm channel region before doping. (c–f), Illustrations of possible dopant arrangements and expected potentials landscapes for transistors with: nominally non-doped channel (device A) (c), randomly-doped channel with *N*_D_ ≅ 1 × 10^18^ cm^−3^ (device B) (d), selectively-slit-doped channel with intermediate doping concentration, *N*_D_ ≅ 5 × 10^18^ cm^−3^ (device C) (e), and selectively-doped channel within a circular area, with high doping concentration, *N*_D_ > 1 × 10^19^ cm^−3^ (device D) (f).

We fabricated different types of devices (labeled **A**, **B**, **C**, and **D**), with the main difference being the channel doping profile, as illustrated in Fig. 1. All devices are silicon-on-insulator field-effect transistors (SOI-FETs) (Fig. 1a), with an ultrathin channel (5–10 nm) with width below 100 nm (Fig. 1b shows a scanning electron micrograph of a device channel before final oxidation). Device **A** has a nominally-undoped 150-nm-long channel (Fig. 1c). Device **B** has the channel discretely and randomly doped with P donors (Fig. 1d), with a concentration *N*_D_ ≅ 1 × 10^18^ cm^−3^, that is, with an average inter-donor distance, *d*_P-P_ ≅ 10 nm. Device **C** (Fig. 1e) has the central region of the channel selectively doped through a slit-opening across the channel width. For that, a 30-nm-wide slit was opened in a doping-mask oxide layer, separated from the lead extensions by 70-nm-wide non-doped gaps (in which mask oxide is preserved). Final dimensions of the slit and gap are ~50 nm each, mainly as a result of side etching of the mask oxide. Lateral diffusion of dopants was suppressed by minimizing the thermal budget during the only subsequent thermal process, gate oxide formation, using a low-temperature wet thermal oxidation (see Methods). Doping concentration was larger for these devices (*N*_D_ ≅ 5 × 10^18^ cm^−3^) in order to promote the formation of “clusters” containing a few coupled donors. For such relatively high concentration, “clusters” of at least 3 donor atoms, with the donor atoms located at distances closer than two Bohr radii from each other (that is, relatively strongly coupled), are prevalent^14^. Assuming a Poisson distribution of dopants, the probability to find 3-donor “clusters” is 17%. Thus, for a 50 nm × 50 nm × 10 nm volume of a typical doped-slit, there will be at least one such multiple-donor “cluster”. In contrast, device **D** (Fig. 1f) was specifically designed to highly favor the formation of a unique multiple-P-donor “cluster” working as a single QD. Doping concentration was purposely increased (*N*_D_ > 1 × 10^19^ cm^−3^) in order to avoid the formation of isolated “clusters” and to increase coupling between the P-donors in the QD. The channel of this device was also selectively doped, but using a circular opening with a diameter of 10–30 nm in the doping-mask oxide, separated from the leads by wider non-doped gaps. It can be evaluated that there are several tens of P-donors coupled to form a QD in device **D**.

### Low-temperature I-V characteristics

**Figure 2:**
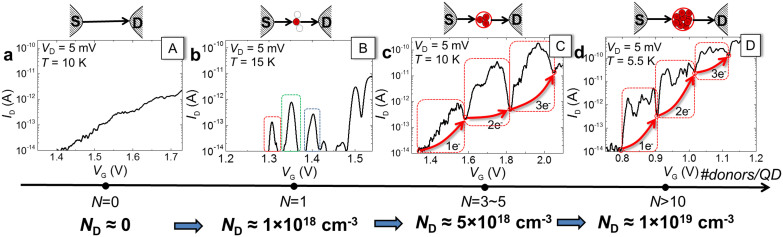
Impact of channel doping on the electrical characteristics. *I*_D_ - *V*_G_ characteristics (*V*_D_ = 5 mV, *T* = 5.5–15 K) measured for SOI-FETs with different channel doping profiles: (a), nominally non-doped channel (device A); (b), randomly, discretely-doped channel (*N*_D_ ≅ 1 × 10^18^ cm^−3^) (device B). Each *I*_D_ peak is ascribed to single-electron tunneling via different P donors, as schematically shown in the inset for one P-donor working as a QD; (c), selectively-slit-doped channel (*N*_D_ ≅ 5 × 10^18^ cm^−3^) (device C). Arrows mark the addition of electrons one by one between consecutive peak envelopes, corresponding to tunneling through a few-P-donor “cluster”-QD, as shown in the inset; (d), selectively-doped channel, doped within a circular area (*N*_D_ > 1 × 10^19^ cm^−3^). Consecutive current peaks are ascribed to tunneling through a many-donor unique QD, as illustrated in the inset. The data is arranged along an axis illustrating the evolution of doping concentration, *N*_D_, and the number of P-donors likely to form the transport-QD.

We measured the electrical characteristics for several devices of each type, at low temperatures (*T* ≤ 15 K), according to the procedure described in Methods. Figure 2 shows, for comparison, representative *I*_D_ - *V*_G_ characteristics, measured for the same source-drain bias, *V*_D_ = 5 mV, for each device type. The data is displayed along an axis reflecting the different doping concentrations, *N*_D_, for each device type, together with the number of P-donors likely to induce the transport-QD.

Device **A** (Fig. 2a) exhibits a monotonous increase of *I*_D_ as a function of *V*_G_, similarly to conventional FETs. A few fine current steps can be observed, which can be ascribed to the effect of quantum confinement on the conduction-band energy states of such an ultrathin channel (5–10 nm thick), as often reported in literature^15^. Device **B** (Fig. 2b), on the other hand, exhibits several isolated *I*_D_ peaks at low *V*_G_'s, before the onset of a larger current. These isolated current peaks, with irregular *V*_G_ periods and *I*_D_ levels, are ascribed to single-electron tunneling (SET) transport via different P donors, each working as a QD (with one P-donor as QD illustrated in the inset)^9^,^11^. This is because each individual P donor can practically accommodate only one electron, except for extremely low temperatures. Device **C** (Fig. 2c), with selectively-doped channel, exhibits a different behavior. As *V*_G_ is increased, several current peak envelopes, with a periodic *V*_G_ spacing, can be identified, separated by current dips (as marked on the graph). These periodic peak envelopes are ascribed to SET transport via a donor-cluster QD that can accommodate several electrons (not only one). Although the background diffusion current is rapidly increased, this interpretation is consistent with the stability diagrams described in the Supplementary Information. Such a donor-cluster QD is formed, with high probability, by a few P donors located close to each other. Multiple-donor QDs have previously been reported to be formed randomly in highly-doped SOI-FETs^16^,^17^,^18^,^19^. For device **C** shown here, since the features embedded in the peak envelopes repeat on consecutive envelopes, it is most likely that only one QD (a unique multiple-donor “cluster”) dominates transport for this low-*V*_G_ range. This is further supported by the results obtained for device **D**, in which a unique QD is favored by the specific doping-pattern design. For a number of devices of this type, as the example shown in Fig. 2d, similar current peak envelopes, with repeated pattern of sub-peaks (inflections) can be found. This suggests that the observed current peak envelopes, as found for both devices of type **C** and **D**, can be assigned to a unique transport-QD. It can also be seen that, for device **D**, a larger number of inflections are found in each current envelope, which is consistent with the effect of a larger number of strongly-coupled P-donors forming a QD in these devices. For device **C**, we evaluate the dimensions of the QD from the *V*_G_ period between consecutive peaks (~200 mV). Assuming a parallel-plate capacitance model, the QD radius is estimated to be approximately 8.5 ± 1.0 nm. This is significantly larger than the Bohr radius for a P donor in bulk^20^,^21^ (*r*_B_ ≅ 2.5 nm), suggesting that the QD covers the area of several P donors.

**Figure 3:**
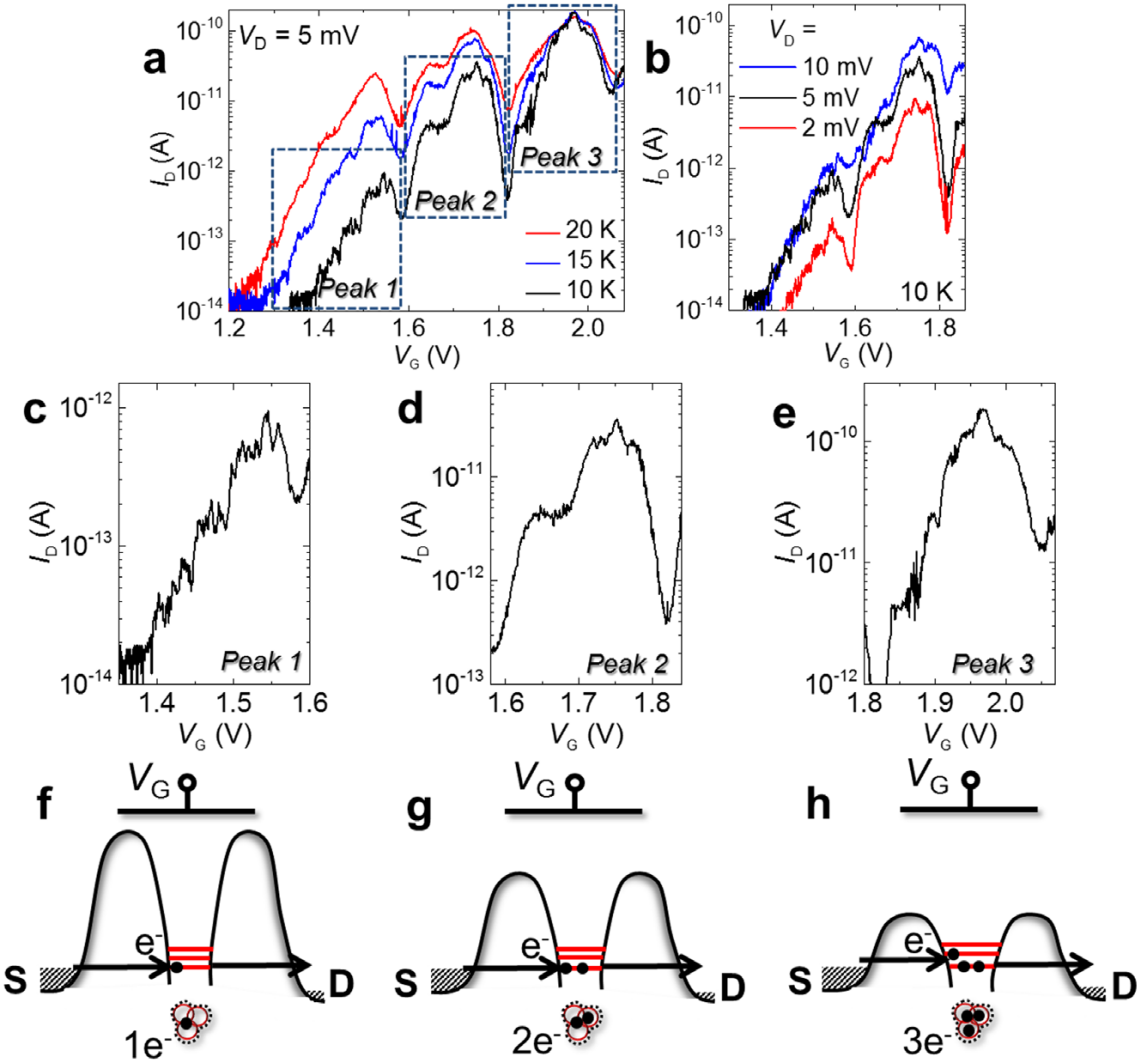
Tunneling transport spectroscopy of a-few-donor QDs formed by selective doping. (a), *I*_D_ - *V*_G_ characteristics (*V*_D_ = 5 mV) measured for several low temperatures (*T* = 10–20 K). Several similar current peak envelopes can be identified. (b), *I*_D_ - *V*_G_ characteristics measured for a few values of *V*_D_, in the range of the first two current peak envelopes. (c), (d), (e), Individual peak envelopes, rescaled for easy comparison. (f), (g), (h), Schematic models of SET transport corresponding to each peak envelope, with the QD formed by a number of 3 P-donors, reflected also in the number of energy levels available for transport (only the ground-state multi-fold is illustrated, since the excited states are expected to be found at significantly higher energies and will not directly contribute in the tunneling transport).

We now turn to a more detailed analysis of the fine features (inflections) observed embedded in each peak envelope for device **C**. In Fig. 3a, *I*_D_ - *V*_G_ characteristics are shown for several low temperatures (10–20 K), which allows us to trace these current inflections. A standard procedure to analyze such features is to monitor their traces in the stability diagrams (plots of *I*_D_ in the *V*_D_-*V*_G_ space). Stability diagrams measured for device **C** are shown in the Supplementary Fig. S1. Since the current peak envelopes appear close or above the onset of diffusion transport, a large diffusion current is superimposed on tunneling transport and the fine features become quickly blurred with increasing *V*_D_. However, it is likely that each current inflection (sub-peak) corresponds to a new energy level of a single QD entering the source-drain bias window. For the shown device, a number of 3–5 such current inflections can be observed.

Figure 3b shows the first two observable current peak envelopes for several values of source-drain bias (*V*_D_) (more examples in the Supplementary Fig. S1). It can be observed that, even from relatively small *V*_D_'s, all inflections (sub-peaks) appear embedded in the envelopes, which suggests that the transport in these devices occurs in a non-linear regime, that is, the transport window is wide enough to contain several energy levels. Our results are, in fact, quite similar also with the results obtained for transport-spectroscopy of single semiconductor-QD in the non-linear transport regime^22^. When a small number of atoms interact with each other, it is known that a multi-fold of energy levels is formed, with the number of split levels practically equal to the number of coupled atoms which form the molecular-like spectrum^23^,^24^. Thus, based on the data obtained for device **C**, shown in this paper, one can evaluate that the QD is formed by 3–5 coupled P donors.

We can estimate the energy separation, Δ*E*, between consecutive levels by converting Δ*V*_G_ (*V*_G_ interval between consecutive inflections) through the lever-arm factor, *α*. *α* is defined as the change in channel potential energy induced by a certain change in *V*_G_. This factor was evaluated^5^ from the analysis of the boundaries of the Coulomb diamonds in stability diagrams (shown in the Supplementary Fig. S1) and from the *V*_G_ dependence of the tunnel barrier height (not shown). The lever-arm factor is found to be *α* ≅ 0.09 ± 0.01 eV/V. Combining this value with typical spacings of Δ*V*_G_ ≅ 20–50 mV leads to energy separations, Δ*E*, in the range of 1.8–4.5 meV. These values are significantly smaller than the energy separation between the ground state and the first excited states for a bulk-like P donor^20^,^21^ (12 meV), being more consistent with expected splitting of the ground-state levels for a few closely-spaced P donors.

According to the above arguments, consecutive *I*_D_ peak envelopes (zoomed-in for comparison in Figs. 3c, d, and e) can be ascribed to different charge states of a QD, changing by exactly one electron between consecutive peak envelopes, as already indicated in Fig. 2. This is also schematically illustrated in the bottom panels shown in Figs. 3f, g, h. As will be discussed later, it is most likely that only the ground-state multi-fold dominantly contributes to transport in these devices, and hence only energy levels corresponding to this multi-fold are illustrated in the shown panels. For low *V*_G_'s, the QD is empty and SET transport takes place by sequential tunneling of electrons one-by-one into the QD from the source and out toward the drain (Fig. 3f). As *V*_G_ is further increased, one electron becomes trapped in the QD, effectively reducing the tunnel barrier height. This leads to an enhancement of the tunnel rate, which may be observed as an increase in the relative *I*_D_ level of the second peak envelope. The trapped electron will most likely occupy the lowest energy level available in the QD, slightly modifying the energy spectrum. The second current peak envelope (Fig. 3d) exhibits some differences, in particular on the left side. However, the top part maintains more similarities, suggesting remaining similarities in the energy spectrum. Further increase of *V*_G_ induces the trapping of a second electron in the QD (Fig. 3g). More noticeable changes can be seen on the third peak envelope (Fig. 3e), for which it is expected that a third electron is added into the QD (Fig. 3h). Based on this interpretation, the fine features embedded in the current peak envelopes are ascribed to tunneling-transport spectroscopy of the energy states of a unique donor-cluster QD.

## Discussion

As described above, the SET transport can reveal information about the number of coupled donors forming a QD when this number is small. In the following, in order to obtain a qualitative picture of the energy spectra, we analyze, by first-principles simulations, silicon nanostructures containing one and a few closely-spaced P-donors. This can allow a qualitative comparison between experimental results and fundamental theory. In this work, the dimensions of the investigated structures (0.5 × 1.6 × 5.0 nm^3^) are much smaller than those of the experimental devices due to limitations of computational time resources, so further optimization is needed before attempting a full quantitative comparison. However, this fundamental approach can provide key insights into the behavior of isolated and coupled donor atoms in nanoscale.

**Figure 4:**
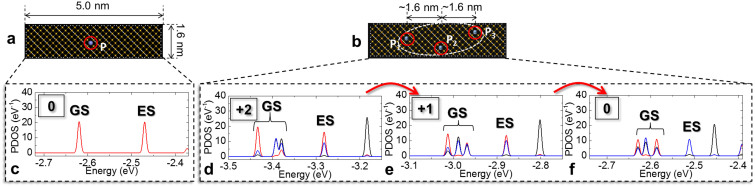
First-principles analysis of nanostructures containing one and multiple P donors. (a), (b), Atomistic views of the simulated nanostructures, containing either one (a) or three (b) P donors (distance between P_1_–P_2_ and P_2_–P_3_ is ~1.6 nm, and between P_1_–P_3_ ~3.0 nm). (c), Projected density-of-states (PDOS) spectra at the P donor location for neutral system (one electron added to the P donor, that is “0” effective charge state). (d), (e), (f), PDOS spectra at all P donor locations (different colors indicate the location of different P-donors), illustrating the splitting of the donors' GS into a GS multi-fold with three distinct levels, well separated in energy from the first excited state (ES). Different charge states of the multiple-donor system are shown as labels for the degree of ionization of the system: (d), +2; (e), +1; (f), 0. These consecutive charge states correspond to successive addition of electrons, one by one, into the multiple-donor system (as indicated by arrows).

We treat atomically-defined nanostructures containing different number of P donors, placed in substitutional positions within the Si matrix, as shown in Fig. 4. In Fig. 4a, we show the structure containing a single P donor, while in Fig. 4b, we show the same structure, but containing three P donors close to each other (inter-donor distances are ~1.6 nm). These two cases correspond to the experimental cases of an isolated P-donor and a few “clustered” P donors, respectively. Projected density of states (PDOS) spectra at the P-donor locations are shown in the lower panels, for the cases of a single P-donor (Fig. 4c) and of three closely-spaced P-donors (Figs. 4d–f); for the case of the triple-P-donor nanostructure, the PDOS calculated at the location of each P-donor is indicated by different colors. Each PDOS peak corresponds to an energy level. We proceed with our analysis by adding electrons one by one into the system of fully-ionized P donor(s) and monitoring the PDOS spectrum as a function of the charge state of the system; for the triple-P-donor system, the “+2” label (Fig. 4d) indicates the condition of doubly-ionized system (that is, with only one electron added into the initially fully-ionized system), the “+1” label (Fig. 4e) indicates the singly-ionized system, and the “0” label (Fig. 4f) corresponds to the neutral system.

For the case of the single-donor nanostructure (Fig. 4a), from the PDOS spectrum shown in Fig. 4c, it is possible to identify the ground state (GS) and the first excited state (ES) of the donor. These levels are well separated from each other, much more than for bulk P donors^20^,^21^, due to the low-dimensionality of the system. Thus, it is likely that only the donor's GS contributes to transport in such single-P-donor channels under typical bias conditions.

In the triple-donor case (Fig. 4b), for all charge states (as illustrated in Figs. 4d–f) the GS is clearly split into a triple-level multi-fold. This is consistent with our expectation that the number of split levels (within the donors' GS multi-fold) can be directly correlated with the number of strongly-coupled donors, at least for the cases containing a limited number of coupled P-donor-atoms. It can also be seen that the distribution of energy levels within this GS multi-fold slightly changes between Figs. 4d, e, and f, while the energy value increases periodically with changing the charge state of the system. These results can be qualitatively correlated with the experiments, in which periodic current peak envelopes exhibit also slight changes of the distribution of the fine current features for different charge states of the multiple-donor QD. Finally, it is worth to note that the excited state (ES) multi-fold remains significantly separated in energy from the GS multi-fold. Mixture of GS and ES multi-folds occurs when the inter-donor distances become extremely small (<1.0 nm), which is not the case of our experimental devices (with average inter-donor distances of ~5.0 nm). Hence, it can be concluded that the transport characteristics only reflect contributions from the GS multi-fold, offering thus a simple way to evaluate the number of strongly-coupled donors.

However, since dopant distribution in the selectively-doped area should have statistical fluctuations, more complex tunneling transport through multi-QDs cannot be excluded. Such a complex transport may also lead to some fine structures within each current peak envelope^25^,^26^. Further experiments may provide more insights and, based on the present study, open new possibilities for designing dopant-based functionalities^27^,^28^,^29^.

In summary, we fabricated, with good controllability, nano-transistors with a QD formed by a few closely-spaced P donors. These strongly-coupled P-donors form an energy spectrum with the ground state multi-fold having a number of energy levels in good correlation with the number of coupled donors, as suggested by first-principles simulations. In this way, low-temperature *I*_D_ - *V*_G_ measurements can offer transport-spectroscopy information about coupling within small cluster-QDs containing a few donor atoms. Further optimization of the nanoscale selective-doping process used in this work may provide a breakthrough for practical applications that would fully utilize quantum and atomic properties of coupled dopant atoms for the implementation of new electronic functionalities.

## Methods

### Device fabrication and structure

Samples were fabricated from silicon-on-insulator (SOI) wafers, with a 150-nm-thick buried oxide (BOX) layer. All processes have been carried out in a clean room environment, using CMOS-compatible techniques. All types of devices (types **A**, **B**, **C**, and **D**) were fabricated using similar steps, except for the channel doping processes. Initial SOI thickness was 55 nm, which was thinned down by sacrificial oxidations and usual oxidation processes to a final thickness of *t*_ox_ = 5–10 nm. All patterning processes, including channel patterning, were done using an electron-beam lithography (EBL) technique; channel width was set as one of the parameters (final channel widths are in the ~10–100 nm range). Gate oxide thickness is ~10 nm for all devices. However, for the selectively-doped devices (devices of type **C** and type **D**), gate oxide was formed by wet thermal oxidation (800°C, 15 min) in order to minimize lateral thermal diffusion of dopants after selective doping. Al was used for the gate, source, and drain contacts.

### Measurement setup for electrical characterization

Electrical characteristics were measured with Agilent 4156C and Agilent B1500A precision semiconductor parameter analyzers using variable-temperature prober station. The samples were placed in a high-vacuum chamber for the *I*-*V* measurements, carried out mainly at low temperatures (5.5–20 K) for the measurements shown here. In all measurements, the source electrode was grounded and a bias was applied to the drain electrode (typically, *V*_D_ = 5 mV). The gate voltage (*V*_G_) was swept as a variable parameter. For the selectively-slit-doped FETs (device **C**), substrate Si was used as a back gate and then converted into an effective gate voltage (*V*_G_) using a conversion factor equal to *t*_BOX_/*t*_ox_ = 150 nm/10 nm = 15.

### First-principle simulations

First-principles simulations shown in this paper were performed using an open-source package, OpenMX (http://www.openmx-square.org/), developed at JAIST. This simulation is based on density functional theory (DFT), norm-conserving pseudopotentials, and pseudo-atomic localized basis functions. In atomistic Si nanostructures, single or multiple P-donors substitutionally replace Si atoms in the matrix and the dangling bonds were finally terminated with H atoms. Simulations were performed for different charge states by adding single electrons into the system with all donor electrons initially removed. Total DOS and electron wave function distributions (not shown) were used to identify the donor GS as the *s*-orbital level below the conduction band edge.

## Author Contributions

M.T., D.M., T.M. and A.S. designed the samples and contributed to the fabrication. D.M. and A.S. carried out the I-V measurements. H.M. and L.T.A. carried out the first-principles simulations. All authors commented on the contents of the manuscript. D.M. and M.T. co-wrote the manuscript.

## Supplementary Material

Supplementary Information Supplementary Information
