# Supplementary material for: Transport spectroscopy of coupled donors in silicon nano-transistors
Source: Sci Rep. 2014 Aug 28;4:6219. doi: 10.1038/srep06219 (PMC4147367; doi:10.1038/srep06219)
Supplement: Supplementary Information [file srep06219-s1.pdf]

Supplementary Information

Transport spectroscopy of coupled donors in silicon nano-transistors

Daniel Moraru[1], Arup Samanta[1], Le The Anh[2], Takeshi Mizuno[1], Hiroshi Mizuta[2,3], and Michiharu Tabe[1*]

[1]*Research Institute of Electronics, Shizuoka University, 3-5-1 Johoku, Naka-ku, Hamamatsu 432-8011, Japan*

[2]*School of Materials Science, Japan Advanced Institute of Science and Technology, 1-1 Asahidai, Nomi 923-1292, Japan*

[3]*Nano Research Group, ECS, Faculty of Physical Sciences and Engineering, University of Southampton, Highfield, Southampton SO17 1BJ, United Kingdom*

Stability diagrams for selectively-doped SOI-FETs (device C)

A usual procedure to identify the origin of the fine features (e.g., inflections) in current peaks due to tunneling transport through a QD is to measure and analyze the stability diagrams, i.e., plots of I_D versus V_G and V_D.[1-3] Such analysis has also been applied to characterize individual donors working as QDs in silicon devices.[4-6] In such diagrams, it is usual to observe zero-current stable-charge regions (Coulomb diamonds) as signatures of Coulomb blockade transport mechanism.

For device **C** shown in the manuscript, we measured the stability diagrams for a V_G range corresponding to the first two current peak envelopes, as shown in Figs. S1**a** and S1**b**. In the V_G-V_D space, absolute value of I_D [abs(I_D)] is plotted; this allows us to show the contour plot in a logarithmic color scale. Since the peak envelopes are affected by relatively large background diffusion current, the fine features are rapidly masked as V_D increases. Different cutoff-current levels are, therefore, used in Figs. S1**a** and S1**b** in order to emphasize features appearing around the first and the second current peak envelope, respectively.

First of all, as delineated by solid lines, distinct Coulomb diamonds can be identified (around V_G~1.6 V and ~1.8 V, respectively). These diamonds can be basically ascribed to consecutive stable charge states of a single QD. The diamonds appear slightly separated from each other in V_G, which seems to be inconsistent with classical Coulomb blockade theory for a QD. However, it should be noted that the background diffusion current is continuously increasing after the onset and this induces some distortion in the Coulomb diamonds. In the model of a single QD, it is reasonable to ascribe the features (inflections) observed within the current regions, i.e., between the Coulomb diamonds, to transport influenced by the discrete energy levels of this QD. Dashed lines are drawn as simple guides to illustrate the expected behavior of such features.

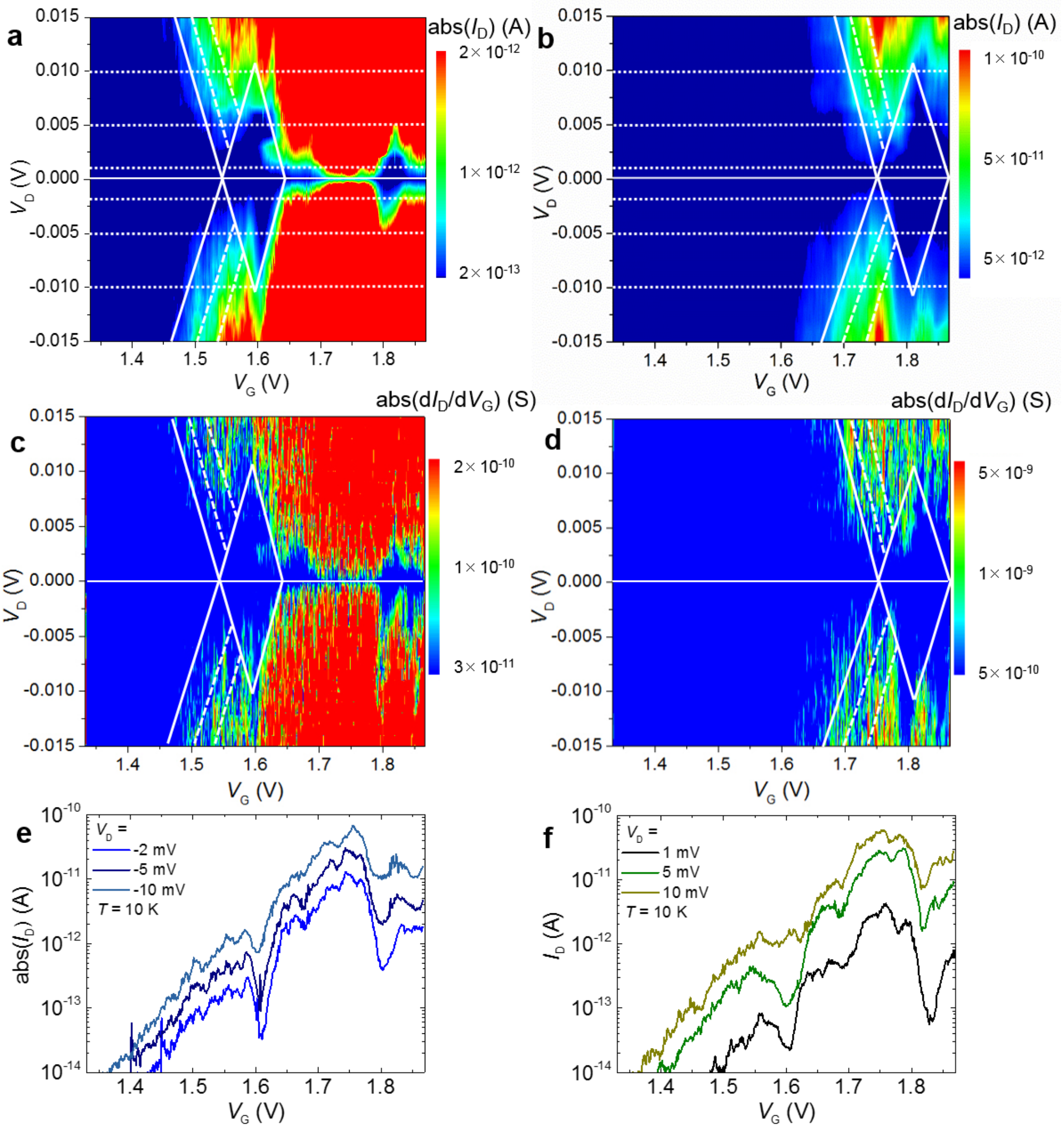

Supplementary Figure S1. Stability diagrams for selectively-doped device. a, b, Stability diagrams (plots of abs(I_D) in the V_D-V_G space) measured at T = 10 K for device **C** shown in the manuscript [in Fig. 2**c** and in Fig. 3]. The current range is adjusted differently to emphasize the features embedded in the first and second current peak envelope, respectively: **a,** 200 fA < I_D < 2 pA; **b,** 5 pA < I_D < 100 pA. The boundaries of the stable-charge regions (Coulomb diamonds) are drawn by solid lines. Dashed lines are drawn to illustrate expected traces that may be induced by tunneling transport via discrete energy levels in the transport-QD. **c, d,** Contour plots of transconductance (here, abs(dI_D/dV_G)), with different cutoff levels. **e, f,** I_D-V_G characteristics along several horizontal dotted lines indicated in Figs. S1**a** and S1**b,** for both negative V_D polarity (shown as abs(I_D) in **e**) and positive V_D polarity (**f**).

Because of the superimposed background diffusion current, the fine features embedded in the peak envelopes become quickly blurred as V_D is increased, which hinders their detailed analysis. Despite the blurring and difficulty in analysis of the fine features, the observed Coulomb diamonds (Fig. S1**a** and S1**b**) are in reasonably good agreement with our interpretation.

In Figs. S1**c** and S1**d**, we also show the transconductance, dI_D/dV_G. In principle, such a plot should emphasize more clearly the fine features, but, since the large diffusion current increases the background current, transconductance is also significantly affected. However, some traces running vertically in these diagrams, i.e., weakly dependent on V_D, are faintly seen. Such features may be related to multi-QD tunneling[7], but further experimental study is needed to fully clarify this behavior in our devices.

We also plot a few I_D-V_G curves for negative V_D's (in Fig. S1**e**) and for positive V_D's (in Fig. S1**f**) along the dotted lines indicated in Figs. S1**a** and S1**b**. The features embedded in the peak envelopes can be thus correlated with the features observed within the stability diagrams. Considering the above data, the most likely origin of the features observed in the current peak envelopes is transport through the energy states of a QD located in the device channel, as also described in the manuscript. However, more complex "multi-tunneling" transport through multi-QDs cannot be completely excluded.

Finally, since the slopes of the stable-charge region (Coulomb blockade) boundaries can be associated with the capacitive coupling of the QD to source, drain, and gate, it is possible to extract the lever-arm factor, α, defined as $\alpha = dE_{QD}/dV_G$ (eV/V), i.e., the fraction of V_G used to modify the energy within the QD (E_{QD}). For device **C**, after analyzing these boundaries (delineated by solid lines in Fig. S1), α is found to be 0.09±0.01 eV/V. This value is used to estimate the energy spacing between consecutive energy levels within the QD, as discussed in the manuscript.

References

1. Kouwenhoven, L.P. *et al*., in *Mesoscopic Electron Transport*, edited by L.L. Sohn, L.P. Kouwenhoven, and G. Schön, NATO ASI, Ser. E, vol. 345 (Kluwer, 1997), pp. 105-214.
2. Averin, D.V. & Nazarov, Y.V., in *Single Charge Tunneling: Coulomb Blockade Phenomena in Nanostructures*, edited by H. Grabert and M.H. Devoret (Plenum Press, New York, 1992), p. 217.
3. Foxman, E.B. *et al*. Effects of quantum levels on transport through a Coulomb island. *Phys. Rev. B* **47**, 10020-10023 (1993).
4. Lansbergen, G.P. *et al*. Gate-induced quantum-confinement transition of a single dopant atom in a silicon FinFET. *Nat. Phys.* **4**, 656-661 (2008).
5. Lansbergen, G.P. *et al*. Lifetime-enhanced transport in silicon due to spin and valley blockade. *Phys. Rev. Lett.* **107**, 136602 (2011).
6. Pierre, M. *et al*. Single-donor ionization energies in a nanoscale CMOS channel. *Nature Nanotechnol.* **5**, 133-137 (2010).
7. Golovach, V.N. *et al*. Single-dopant resonance in a single-electron transistor. *Phys. Rev. B* **83**, 075401 (2011).
